# Bradykinin B_2_ receptor blockade and intradialytic hypotension

**DOI:** 10.1186/s12882-023-03192-4

**Published:** 2023-05-11

**Authors:** Jorge L. Gamboa, Cindy A. Mambungu, Adrienne R. Clagett, Hui Nian, Chang Yu, T. Alp Ikizler, Nancy J. Brown

**Affiliations:** 1 Department of Medicine, Division of Clinical Pharmacology, Vanderbilt University Medical Center, 2222 Pierce Avenue 561B-PRB, Nashville, TN 37232-6602 USA; 2 Veterans Administration Tennessee Valley Healthcare System, Nashville, TN USA; 3 Department of Medicine, Division of Nephrology and Hypertension, Vanderbilt University Medical Center, Nashville, TN USA; 4 Department of Biostatistics, Vanderbilt University Medical Center, Nashville, TN USA

**Keywords:** Bradykinin, Hypotension, Hemodialysis

## Abstract

**Introduction:**

Intradialytic hypotension (IDH) is a common clinical complication and is associated with increased morbidity and mortality in patients undergoing maintenance hemodialysis (MHD). The pathogenesis of IDH has been attributed to the rapid reduction of plasma volume during hemodialysis and the inadequate compensatory mechanisms in response to hypovolemia, such as the lack of vasoconstriction. This may be due to the increased production of vasodilators, such as bradykinin. In this study we test the hypothesis that bradykinin B_2_ receptor blockade prevents intradialytic hypotension.

**Methods:**

We performed a post-hoc analysis of a double-blind, placebo-controlled, randomized, 2 × 2 crossover clinical trial comparing the continuous infusion of icatibant, a bradykinin B_2_ receptor blocker, and placebo during hemodialysis. Icatibant or placebo was infused for 30 min before and during hemodialysis in 11 patients on MHD.

**Results:**

Seven of the patients had IDH, defined as a reduction of systolic blood pressure equal to or greater than 20 mmHg during hemodialysis. Stratified analysis, based on the presence of IDH, revealed that icatibant prevented the decrease in blood pressure compared to placebo in patients with IDH [blood pressure at average nadir (2.5 h after hemodialysis): Placebo,114.3 ± 8.9 vs. icatibant, 125.6 ± 9.1 mmHg, mean ± S.E.M]. Icatibant did not affect blood pressure in the group of patients without IDH.

**Conclusion:**

Bradykinin B2 receptor blocker may prevent the occurrence of IDH. Further studies should evaluate the hemodynamic effects of icatibant during hemodialysis and the symptomatology associated with IDH.

**Supplementary Information:**

The online version contains supplementary material available at 10.1186/s12882-023-03192-4.

## Introduction

Patients with end-stage kidney disease (ESKD) on maintenance hemodialysis (MHD) have a decreased life expectancy and increased mortality risk [1, 2]. A plethora of studies also suggests that hemodialysis-associated hemodynamic changes lead to significant adverse effects including patient-reported symptoms such as dizziness, cramps, and fatigue [3, 4].

Intradialytic hypotension (IDH), a common clinical complication of hemodialysis, is defined as a decrease in systolic blood pressure (SBP) of 20 mmHg or more, and it is usually accompanied by symptoms such as cramps, dizziness, headache, and nausea [5]. Approximately 75% of patients on MHD suffer at least one episode of hypotension during hemodialysis, [6] and IDH occurs in approximately 20 to 30% of all regular hemodialysis treatments.[6;7] IDH is also associated with increased morbidity and mortality [8–12].

The pathogenesis of intradialytic hypotension has been attributed to the inappropriately rapid reduction of plasma volume during hemodialysis and the inadequate mechanisms to respond to hypovolemia in patients on MHD [13]. The lack of compensatory vasoconstriction is one of the potential mechanisms associated with intradialytic hypotension. This inadequate response may be due to the removal of vasoconstrictors or the increased production of vasodilators. We and others have shown that hemodialysis induced the activation kallikrein-kinin system and the production of bradykinin [14, 15]. Bradykinin may affect blood pressure during hemodialysis by inducing vasodilation through activation of the bradykinin B_2_ receptor, [16] and contributing to the pathogenesis of intradialytic hypotension.

In this study, we tested the hypothesis that blocking the B_2_ receptor endogenous bradykinin prevents intradialytic hypotension, defined as a drop in systolic blood pressure equal to or greater than 20 mmHg. For this purpose, we conducted a post-hoc analysis of a double-blind, placebo-controlled, randomized, two by two crossover study comparing the effect of continuous infusion of the bradykinin B_2_ receptor blocker icatibant, versus placebo on mitochondrial function during hemodialysis (primary outcome). Blood pressure was defined *a priori* as a secondary outcome of this clinical trial.

## Materials and methods

### Study population

**Fig. 1:**
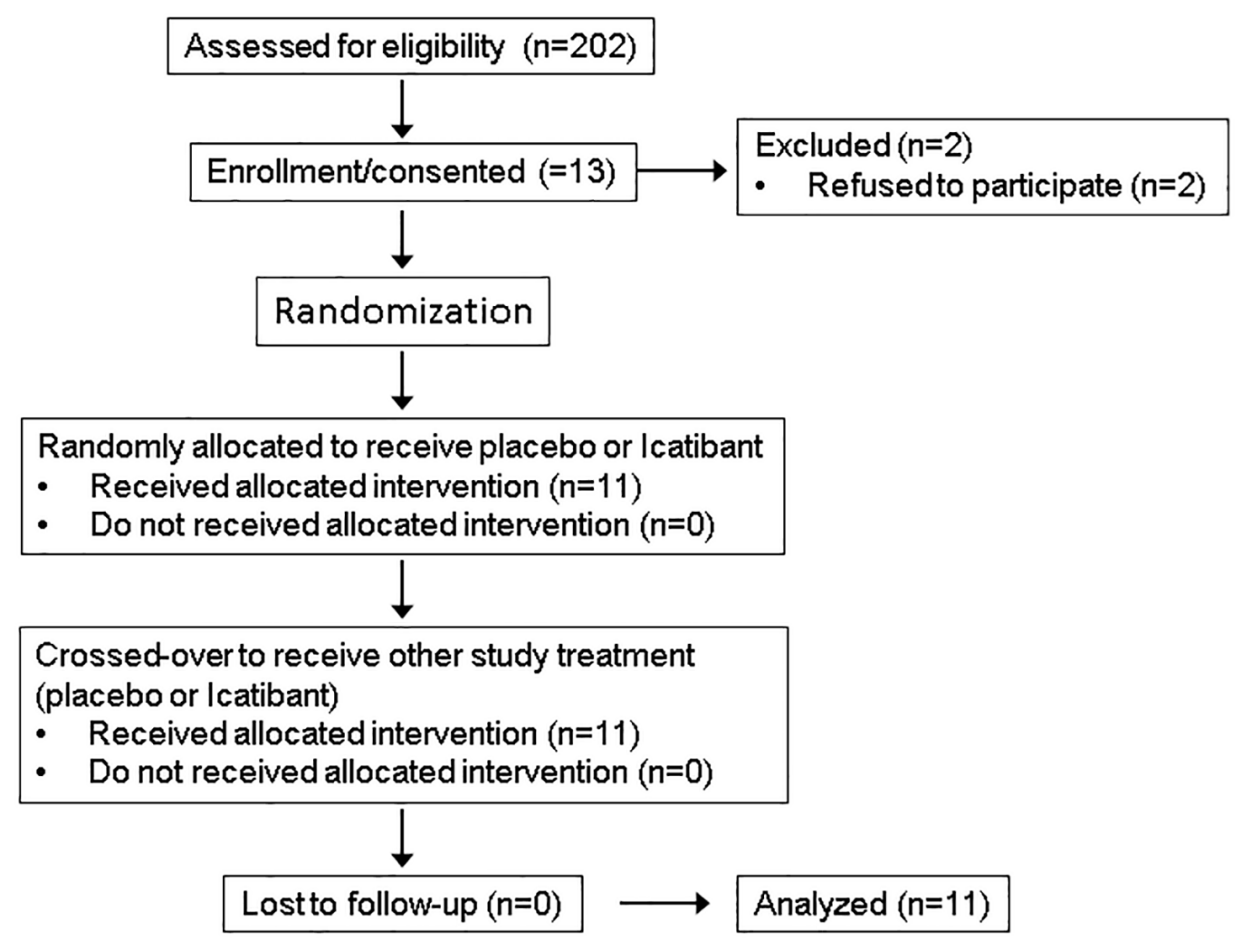
Flowchart of study participation and randomization

The study was approved by the Vanderbilt University Institutional Review Board and performed according to the Declaration of Helsinki. Two-hundred and two patients were pre-screened for eligibility, and 16 patients were approached and assessed for eligibility. Patients with a history of functional transplant less than six months prior to the study or anticipated live donor kidney transplant were excluded. Patients with a history of active connective tissue disease, acute infection within one month prior to the study, advanced liver disease, gastrointestinal dysfunction requiring parental nutrition, or active malignancy were not included in the study. Use of the following medications was also excluded: anti-inflammatory medication other than aspirin at a dose less than 325 mg per day, immunosuppressive drugs within one month prior to the study, vitamin E at a dose higher than 60 IU per day, or vitamin C at a dose higher than 500 mg per day. Subjects with a history of myocardial infarction or cerebrovascular event within three months prior to the study or with an ejection fraction lower than 40% were excluded. Other exclusion criteria were a history of ACEi-associated angioedema or cough, pregnancy, or breastfeeding. Three patients were excluded based on their inability to undergo magnetic resonance imaging (MRI). Thirteen patients provided written informed consent and agreed to participate in the study, but two withdrew from participation before any intervention. Therefore, 11 patients completed the study. Figure 1 shows the flow diagram of patient enrollment. All participants had undergone adequate hemodialysis (Kt/V > 1.2) thrice weekly for at least six consecutive months. Hemodialysis was performed using a Fresenius Optiflux 180 dialyzer (Fresenius Medical Care, Waltham, MA) with a polysulphone membrane. Dialysate quality was within the Association for the Advancement of Medical Instrumentation (AAMI) standards for endotoxin concentrations. In addition, hemodialysis was performed using Diasafe filters (Fresenius Medical Care), to ensure further purity of the dialysate. Patients were clinically stable with pre-hemodialysis potassium levels lower than 6.0 mmol/L.

### Study protocol

**Fig. 2:**
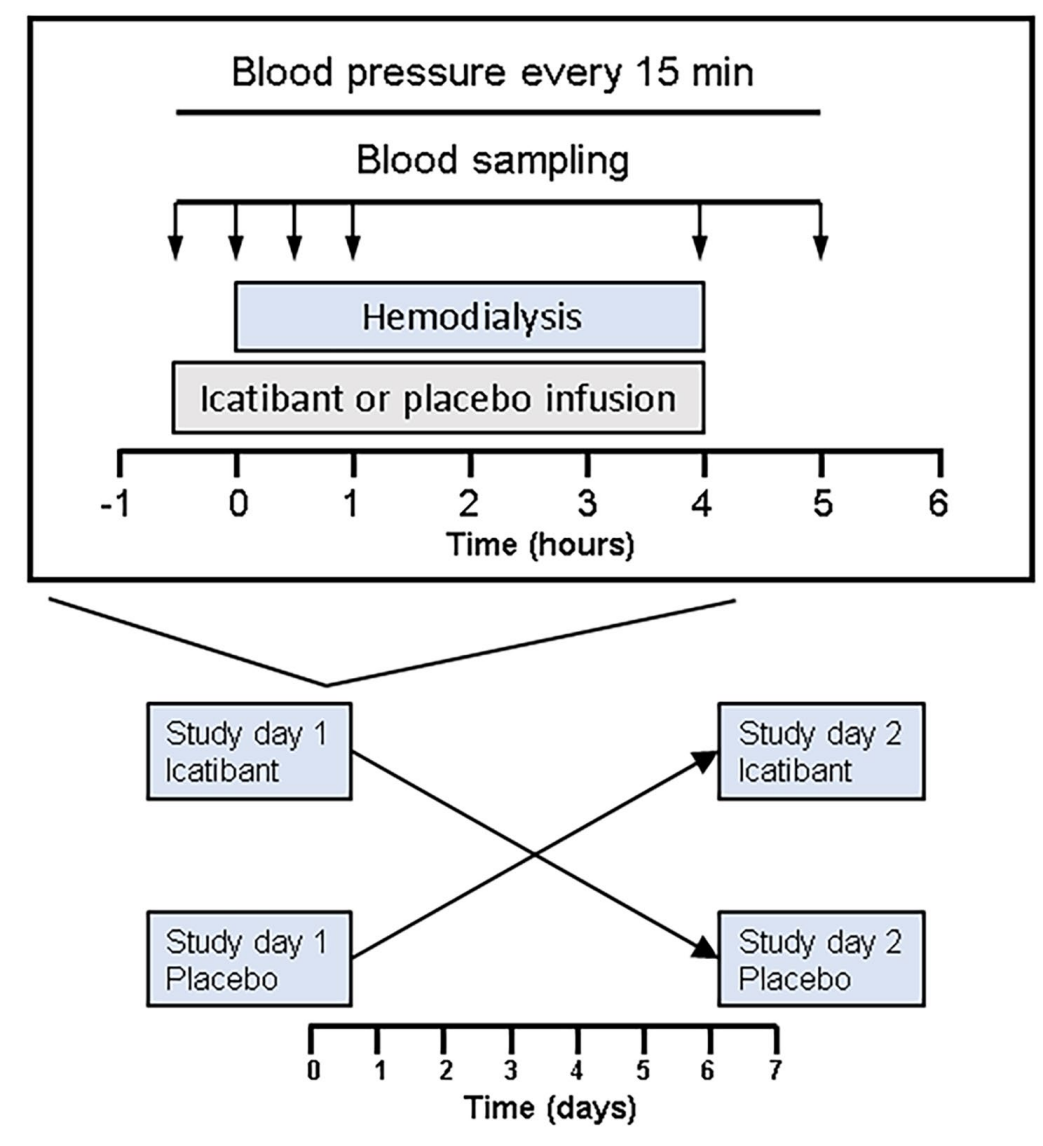
Study design. The study was a randomized, cross-over, double-blind, placebo-controlled study to study the effects of icatibant in mitochondrial function and blood pressure during hemodialysis

The study procedures were performed in the Vanderbilt University General Clinical Research Center (GCRC). Patients participated in a randomized placebo-controlled two by two crossover study, in which they received either, the bradykinin B_2_ receptor blocker icatibant or placebo on the first study day. After a washout period of one week, participants returned to the Vanderbilt GCRC for the second study day to receive the other treatment (Fig. 2). Icatibant (30 mg) was diluted in 0.9% sodium chloride (NS) to a concentration of 100 ug/ ml. Icatibant or placebo (NS) was infused for 30 min prior to the initiation of hemodialysis at a rate of 100 µg/kg/h. The volume of infusion was the same on both study days. At the beginning of hemodialysis, the dose was changed to 50 µg/kg/h for the duration of hemodialysis. Previous studies have found that this dose of icatibant blocks the vasodilator effect and the tissue plasminogen activator release in response to infused bradykinin.[17;18] Subjects received their usual hemodialysis prescription, including the duration, for both study visits. Blood pressure was measured every 15 min during the duration of the study in the contralateral side of the arterial-venous access. Blood samples were collected from the arterial tubing of hemodialysis 30 min before hemodialysis, at the beginning of hemodialysis, 30 min and one hour after the initiation of hemodialysis, at the end of hemodialysis, and one hour after the end of hemodialysis. At the end of hemodialysis, patients remained in the GCRC for two hours; the last blood samples were collected.

Intradialytic hypotension was defined as a reduction of SBP equal to or greater than 20 mmHg during hemodialysis. We restricted the analysis of blood pressure to the first three hours of hemodialysis since the duration of hemodialysis varies from 3.25 to 4.5 h and most episodes of hypotension occurred during the first hour of hemodialysis.

### Laboratory procedures

For bradykinin measurements, blood was collected into polypropylene tubes containing chilled ethanol and let the mixture stand for 30 min at 4 °C. Samples were then centrifuged and the supernatant was stored frozen at -80 until processing. Bradykinin was measured by high-perfomance liquid chromatography (HPLC) following by electrospray inoisation mass spectrometry (ESI-MS) as previously described [19].

### Statistical methods

Data are presented as mean ± SEM or median and interquartile range. We also estimated the area under the curve for the change from baseline over time in blood pressure and heart rate, calculated by summing the numerical values of successive linear segments for every 15 min intervals. We used McNemar’s test to compare the occurrence of intradialytic hypotension between the treatment groups. The effect of treatment on blood pressure and heart rate were analyzed by comparing the areas under the curves with the Wilcoxon paired test.

## Results

### Baseline patient characteristics

Baseline characteristics are shown in Table 1. Causes of ESKD were hypertension (seven patients), lupus glomerulonephritis (one patient), immune-complex glomerulonephritis (one patient), focal segmental glomerulosclerosis (one patient), and chronic urinary tract obstruction due to posterior urethral valves (one patient).

**Table 1:** Baseline characteristics

Parameter		Value
**Age, (years)**		44.73 ± 10.65
**Gender, N male (%)**		8/11 (72.7%)
**Race, N African American (%)**		8/11 (72.7%)
**Smoker, N (%)**		4/11 (36.4%)
**BMI, kg/m** ^**2**^		29.51 ± 7.90
**History of hypertension, N (%)**		11/11 (100%)
**ACE inhibitor or ARB use, N (%)**		1/11 (9%)
**Glucose, mg/dL**		86.91 ± 12.54
**Creatinine, mg/dL**		12.35 ± 4.30
**Hemoglobin, %**		11.50 ± 1.89
**Albumin, g/dl**		4.15 ± 0.39
**Calcium x phosphate product, mg** ^**2**^ **/dL** ^**2**^		43.81 ± 13.48

ACE, angiotensin-converting enzyme; ARB, angiotensin receptor blocker. Data are presented as mean ± SD

### Effect of hemodialysis and bradykinin receptor blockade on blood pressure

Icatibant treatment did not affect blood pressure during hemodialysis compared to placebo treatment in the overall group (Figure S1).

We next analyzed the effect of icatibant on blood pressure in patients with and without intradialytic hypotension.

**Fig. 3:**
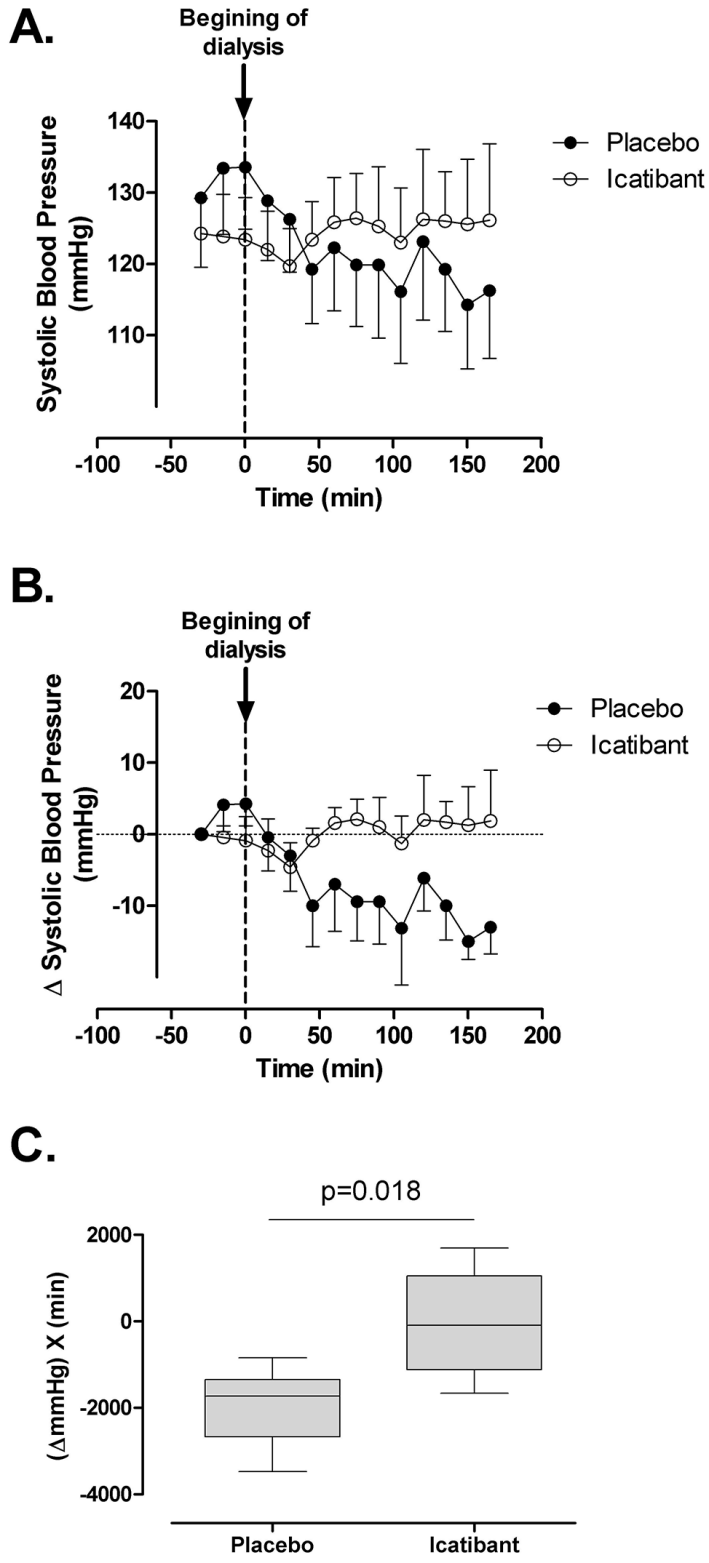
Effect of icatibant on systolic blood pressure (SBP) during hemodialysis in patients with a reduction of blood pressure ≥ 20 mmHg (n = 7). **A.** SBP was monitored every 15 min before and during hemodialysis. **B** and **C**, change in blood pressure from baseline and area under the curve showing that icatibant prevented the reduction in blood pressure during hemodialysis

The hemodialysis variables in patients with and without intradialytic hypotension are shown in Table 2. In patients with intradialytic hypotension during placebo treatment, icatibant prevented the decrease in systolic blood pressure observed during placebo (Fig. 3). There was a similar trend of icatibant on mean arterial blood pressure and diastolic blood pressure (Figure S2). In addition, of the seven patients who developed intradialytic hypotension during the placebo administration, only one patient experienced hypotension during icatibant treatment (p = 0.031, Table 3).

**Table 2:** Hemodialysis variables in patients with and without intradialytic hypotension

Variables	IDH (N = 7)	No IDH (N = 4)	p value
spKt/V	1.51 ± 0.04	1.48 ± 0.18	0.9
Time of treatment (minutes)	241.4 ± 33.8	232.5 ± 8.7	0.5
Specific ultrafiltration rate (ml/kg/h)	9.80 ± 2.64	9.81 ± 4.2	0.9
Ultrafiltration volume	3.68 ± 1.85	2.70 ± 1.07	0.3
Specific ultrafiltration volume (mL/kg)	40.03 ± 14.51	37.88 ± 15.70	0.8
Ultrafiltration rate (L/h)	0.88 ± 0.33	0.70 ± 0.28	0.4
Arteriovenous access type, N (%)			
Fistula	1 (86)	2 (50)	
Graft	6 (14)	1 (25)	
Catheter		1 (25)	
Dialysis vintage, months	394 (103, 852)	155 (43, 589)	0.2
Medications, N (%)			
ACEi/ARB		1 (25)	
CCB	2 (29)	2 (50)	
Beta-blockers	2 (29)	3 (75)	

Variables are expressed as mean ± SD, median (range), unless otherwise specified. IDH, intradialytic hypotension (decrease in systolic blood pressure equal to or greater than 20 mmHg). spKt/V, single pool Kt/V. ACEi, angiotensin-converting enzyme inhibitors; ARB, angiotensin receptor blockers; CCB, calcium channel blockers

**Fig. 4:**
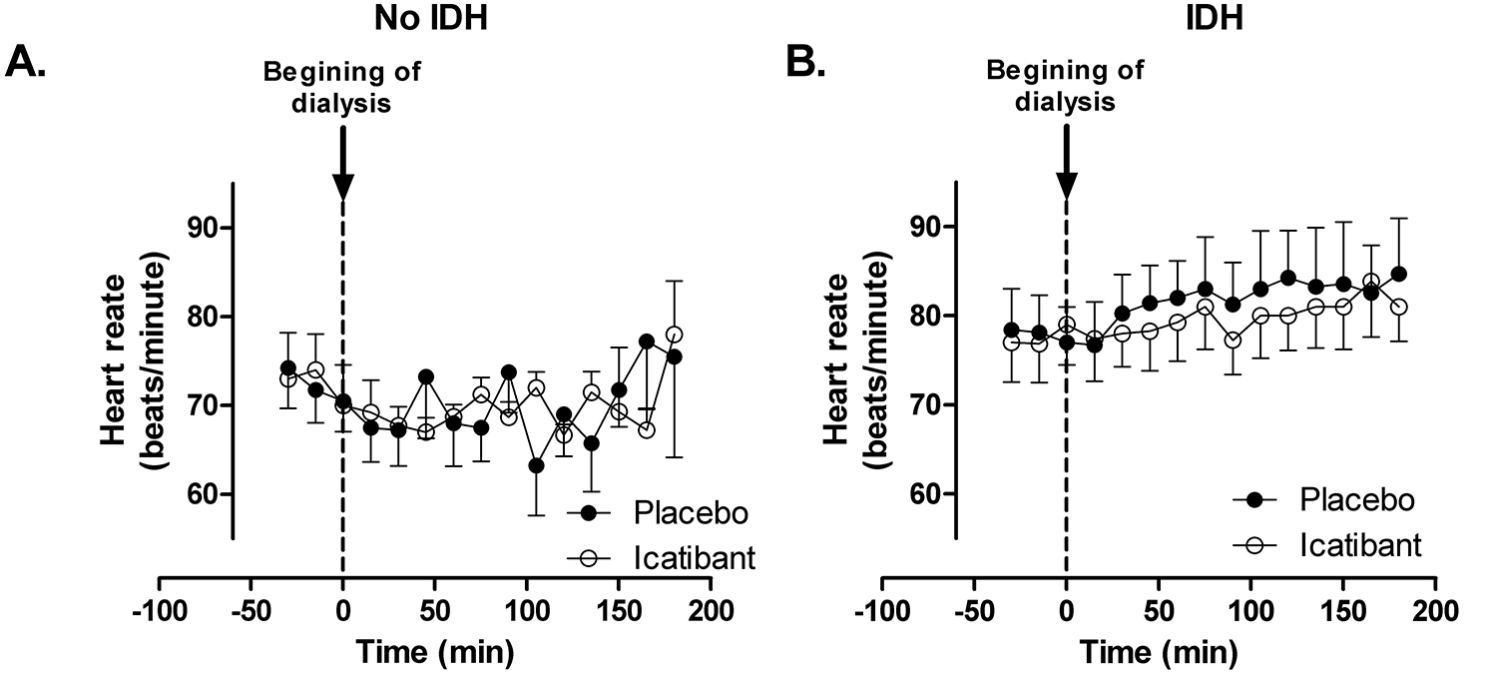
Effect of icatibant on heart rate during hemodialysis in patients without (n = 4, A) and with (n = 7, B) intradialytic hypotension (IDH, a reduction of blood pressure ≥ 20 mmHg). Heart rate was measured every 15 min before and during hemodialysis

**Table 3:** Episodes of intradialytic hypotension during icatibant and placebo treatments

		IDH	No IDH	
Placebo		7	4	
Icatibant		1	10	

IDH, intradialytic hypotension (decrease in systolic blood pressure equal to or greater than 20 mmHg).

Icatibant did not affect heart rate during hemodialysis in patients with intradialytic hypotension (Fig. 4), although the change in heart rate change over the first 30 min after the initiation of hemodialysis was greater in patients with intradialytic hypotension than in patients without (1.9 ± 1.6 versus − 7.0 ± 4.4, p = 0.02). Icatibant did not affect blood pressure in patients without intradialytic hypotension.

### Effect of icatibant on bradykinin concentrations

Bradykinin concentrations were measured prior to, at the beginning of hemodialysis, at 30, and 60 min after initiation of hemodialysis. There was no effect of icatibant versus placebo on bradykinin levels during hemodialysis. In addition, patients who experienced intradialytic hypotension had similar bradykinin levels compared to patients who did not experience hypotension [80.9 (37.5, 104.8) vs. 85.9 (45.0, 155.1) fmol/ml, median (interquartile range), at 30 min, p = 0.66].

### Adverse events

There was no adverse event related to the study intervention. Three patients delevop symptoms associated with hypotension. All of them reported cramps and one of them also complain of dizziness.

## Discussion

In this pilot study, we found that bradykinin B_2_ receptor blockade with icatibant during hemodialysis prevents intradialytic hypotension, suggesting that endogenous bradykinin contributes to intradialytic hypotension. While we are not able to define the primary mechanism, our findings suggest that interfering with the vasodilatory effect of bradykinin during hemodialysis could be useful in those patients with a predisposition to intradialytic hypotension.

The lack of vasoconstriction in response to hypovolemia is one of the impaired responses during intradialytic hypotension. Previous studies suggest that vasopressin, nitric oxide, and adenosine are associated with the occurrence of intradialytic hypotension [20–25]. A recent study also showed that blockade of adenosine A_1_ receptor may prevent the occurrence of hypotension during hemodialysis [26].

Bradykinin is a potent vasoactive peptide that results from the cleavage of high molecular kininogen by tissue and plasma kallikreins and causes vasodilation through the activation of the bradykinin B_2_ receptor [16]. Hemodialysis increases bradykinin levels by activating the kallikrein-kinin cascade and the contact system [14, 27]. Bradykinin is also increased during cardiopulmonary bypass surgery and contributes to the hypotensive response to protamine [28, 29]. Similar to our results in patients undergoing hemodialysis, icatibant attenuates these hypotensive events during cardiopulmonary bypass [29].

Intradialytic hypotension is a common clinical complication of hemodialysis, and it is associated with increased morbidity and mortality [5, 9, 11, 30, 31]. It has been shown that intradialytic hypotension may induce myocardial dysfunction due to reduced myocardial blood flow [12]. Recurrent episodes of hypotension are also associated with frontal lobe atrophy in the brain, [8] and with mesenteric ischemia [32]. Intradialytic hypotension is also associated with common patient-reported symptoms, particularly dizziness and cramps [3]. Furthermore, intradialytic hypotension and a higher specific ultrafiltration rate have been associated with longer hemodialysis recovery time, [33] a measure of quality of life (QOL) that has been validated in patients on hemodialysis that correlates well with longer and more complex QOL questionnaires [34]. Thus, future studies should evaluate the effect of icatibant not only in blood pressure during hemodialysis but also on alleviating the associated patient-reported outcomes.

Bradykinin usually peaks within the first 15 min of hemodialysis, [14] which may explain why we did not observe an increase in bradykinin levels during hemodialysis. This may also explain why we did not detect a difference in bradykinin levels between patients who experienced intradialytic hypotension and patients who do not develop hypotension. Another explanation may reside in individual genetic susceptibility to bradykinin. It has been shown that a particular genetic polymorphism of the bradykinin B_2_ receptor gene (BE1) is associated with bradykinin-induced vasodilation [35]. This susceptibility may also explain the lack of effect of icatibant on blood pressure in patients without intradialytic hypotension. Future studies should evaluate the relationship between polymorphisms of the bradykinin B_2_ receptor gene and the risk of intradialytic hypotension.

We have not measured the clearance of icatibant by the dialyzer, however, vitamin B12 has similar biophysical properties compared to icatibant, such as molecular weight and water solubility. In this study, the KoA (dialyzer mass transfer area coefficient) for vitamin B12 was 312 ml/min (at blood and dialysate flow of 300 and 500 mL/min, respectively). Thus, some amount of icatibant may be cleared during hemodialysis and limit its effectiveness. For this reason, icatibant was continuously infused before and during hemodialysis. Nevertheless, further studies should evaluate the clearance of icatibant during hemodialysis.

This study has some limitations: First, our study should be considered exploratory since our inclusion criteria did not specifically select patients with a documented history of hemodialysis-induced hypotension, and the primary objective of the study was to study mitochondrial function and blood pressure during hemodialysis, but not intradialytic hypotension. While our results are intriguing, future studies should target patients with a history of dialysis-induced hypotension to confirm our findings. Second, this study included young, predominantly male, and African American patients. Thus, the results of the study may not be generalized to a more diverse population of patients on hemodialysis. There were also numerous exclusion criteria that may limit the generalization of the results. Finally, while we used a cross-over design, our sample size is relatively small, and our results should be interpreted with caution.

In conclusion, our study suggests that endogenous bradykinin contributes to the decrease in blood pressure during hemodialysis. Pharmacological therapies that block the effect of bradykinin or inhibit the activation of the kallikrein-kinin system, such as the ones approved for use in hereditary angioedema, represent potential novel treatments for the prevention of intradialytic hypotension.

## Electronic supplementary material

Below is the link to the electronic supplementary material.

Supplementary Material 1

## Data Availability

The data that support the findings of this study are included in the article and supplementary material. Further inquiries can be directed to the corresponding author.
