# Supplementary material for: Bradykinin B2 receptor blockade and intradialytic hypotension
Source: BMC Nephrol. 2023 May 11;24:134. doi: 10.1186/s12882-023-03192-4 (PMC10176680; doi:10.1186/s12882-023-03192-4)
Supplement: Supplementary file 1 — Supplementary Material 1 [file 12882_2023_3192_MOESM1_ESM.pdf]

Supplementary Figure 1.

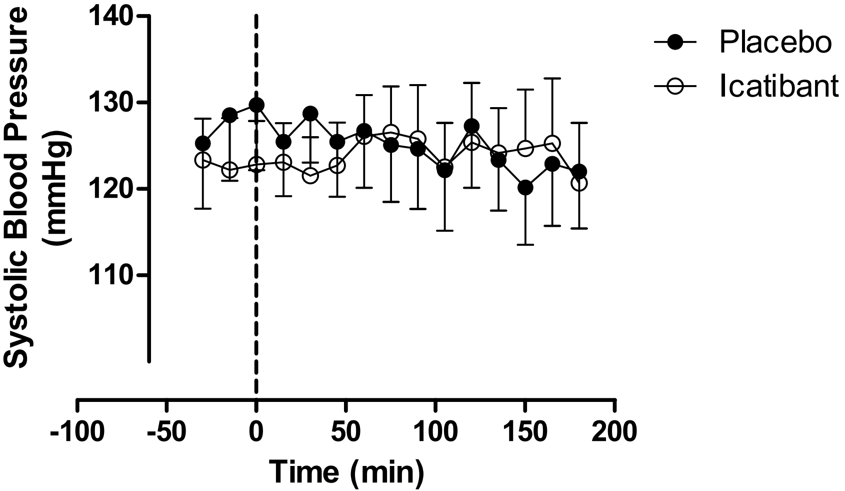

Effect of icatibant on systolic blood pressure during hemodialysis in all the evaluated patients (n=11).

Supplementary Figure 2

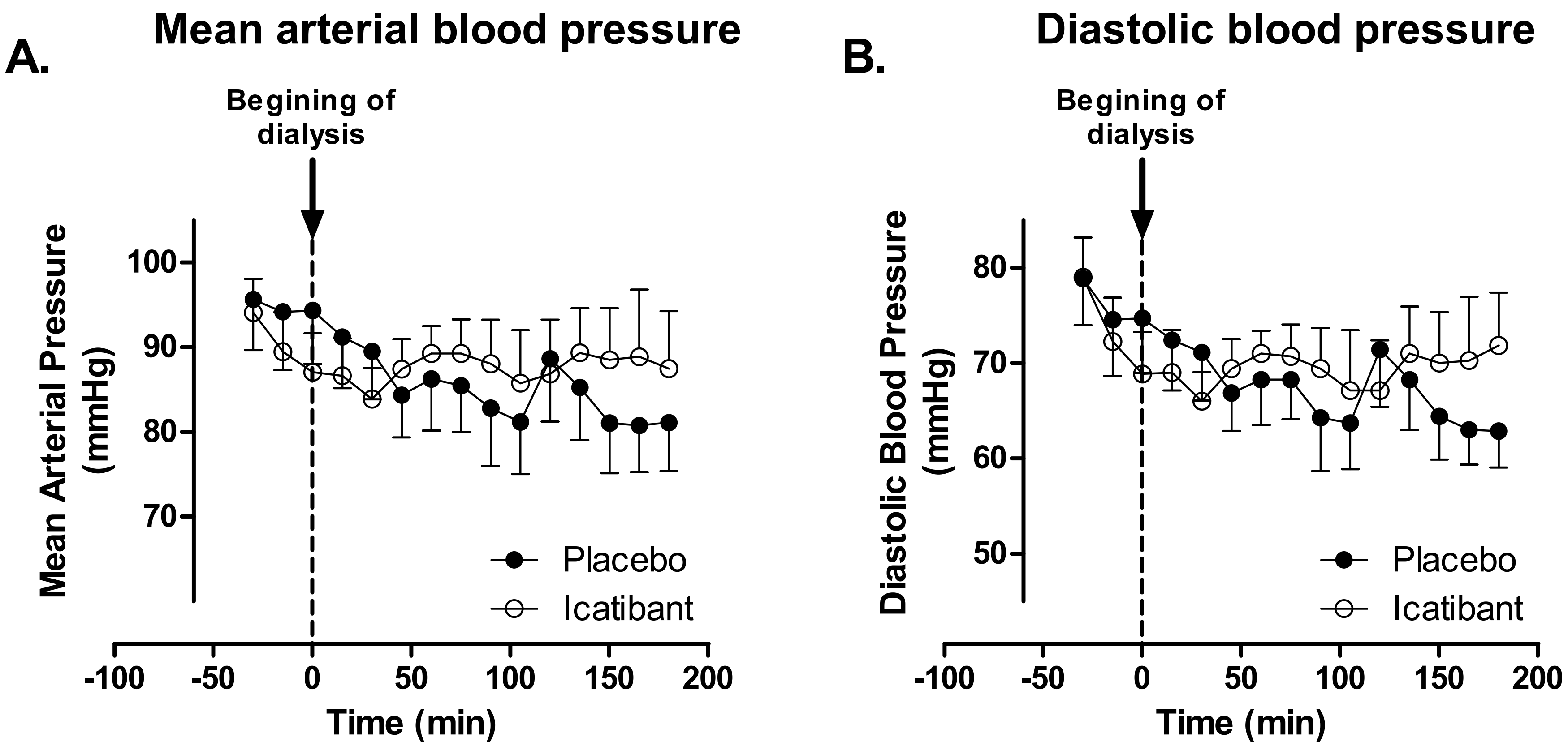

Effect of icatibant on mean arterial blood pressure (**A**) and diastolic blood pressure (**B**) during hemodialysis in patients with a reduction of blood pressure ≥ 20 mmHg (n=7).
